# Fish Skin Mucus Extracts: An Underexplored Source of Antimicrobial Agents

**DOI:** 10.3390/md21060350

**Published:** 2023-06-07

**Authors:** Rocío Díaz-Puertas, Mikolaj Adamek, Ricardo Mallavia, Alberto Falco

**Affiliations:** 1 Institute of Research, Development and Innovation in Healthcare Biotechnology in Elche (IDiBE), Miguel Hernández University, 03202 Elche, Spain; r.diaz@umh.es (R.D.-P.); r.mallavia@umh.es (R.M.); 2 Fish Disease Research Unit, Institute for Parasitology, University of Veterinary Medicine, 30559 Hannover, Germany; mikolaj.adamek@tiho-hannover.de

**Keywords:** marine organisms, fish, skin mucus, extract, antimicrobial, antibacterial, antifungal, antiviral, omics

## Abstract

The slow discovery of new antibiotics combined with the alarming emergence of antibiotic-resistant bacteria underscores the need for alternative treatments. In this regard, fish skin mucus has been demonstrated to contain a diverse array of bioactive molecules with antimicrobial properties, including peptides, proteins, and other metabolites. This review aims to provide an overview of the antimicrobial molecules found in fish skin mucus and its reported in vitro antimicrobial capacity against bacteria, fungi, and viruses. Additionally, the different methods of mucus extraction, which can be grouped as aqueous, organic, and acidic extractions, are presented. Finally, omic techniques (genomics, transcriptomics, proteomics, metabolomics, and multiomics) are described as key tools for the identification and isolation of new antimicrobial compounds. Overall, this study provides valuable insight into the potential of fish skin mucus as a promising source for the discovery of new antimicrobial agents.

## 1. Introduction

The windfall for human and animal health in terms of effectively fighting infectious diseases is being threatened by the resurgence and appearance of dangerous pathogens, which largely outpace the discovery and implementation of new antimicrobials. Growing resistance to current antibiotics [1], antifungals [2], and antivirals [3] is one of the greatest reasons for this. This situation is substantially worsened by the long-lasting drought (with a few recent exceptions [4]) in the discovery of new classes of antibiotics since 1962 [1,5] and the continued scarcity and specificity of antifungals [6] and antivirals [7]. On top of that, factors including population growth, intensive farming, globalization, pollution, and climate change are also contributing notably to this issue by negatively unbalancing the pathogen–host–environment interplay [8].

Thus, in order to address the public health menace posed by both new and “renewed” infectious diseases that are quite often unfortunately associated with considerable morbidity and mortality, it is crucial to expand the arsenal of antimicrobials. This is because, even in an unfavourable scenario of rapid generation of antimicrobial resistance, their availability would at least help to buy time for the development of other countermeasures, such as effective vaccines. In this regard, different antimicrobial search and development strategies with high expectations are being adopted [9,10,11]. However, this is not an easy task.

To paraphrase Spellberg et al., (2007) [1], in this war against microorganisms, humanity has definitely underestimated the power of an enemy army that outnumbers, outweighs, and out-experiences us by several orders of magnitude in all its main divisions (viruses, bacteria, and fungi) (Table 1) [12,13,14]. Furthermore, as mentioned above, society is currently running out of effective ammunition, i.e., antimicrobials, because they have become obsolete in the face of today’s new needs. In this sense, and particularly referring to antibiotics, it should not be disregarded that most of the known antibiotics, as well as their resistance determinants, already existed in nature since ancient times, and humans only discovered them [15]. Indeed, most of them are secondary metabolites or their synthetic derivatives (i.e., natural or semisynthetic antibiotics) that originated primarily from microorganisms, among which the actinobacteria of the genus *Streptomyces* stands out as having provided about two-thirds of the natural antibiotics currently used clinically [16]. This should not discourage rational design efforts to expand the repertoire of synthetic antibiotics that, while still small (e.g., azoles, sulfones, ethambutol, nitrofurans, phenazines, quinolones, and thioamides), have already shown promise in terms of activity and safety [17,18]. However, there is no doubt that the search for natural antimicrobials in biological allies with far more combat experience against these enemies should continue, and that screening capabilities should be improved by exploiting existing and new technologies [11].

## 2. Fish Skin Mucus as a Promising Source of Antimicrobials

In this context, marine ecosystems still remain an option with great potential for the discovery of new compounds, as they are relatively unexplored in this regard. Furthermore, they are the most extensive and ecologically diverse ecosystems, and, therefore, harbor the largest biological and hence biochemical diversity on the planet [28,29,30,31]. As expected, in this highly competitive environment [26,28], microorganisms are currently the fastest growing group of marine producers from which new compounds and antimicrobials are being discovered [32,33].

However, the contribution of higher counterparts, such as algae, plants, and animals, has been, and still is, particularly important [32,34], even considering that many compounds initially attributed to them may actually belong to associated or symbiotic microorganisms [32,35]. Among the animals, the majority of suppliers are invertebrates, mainly (in order of contribution) sponges as the overall top producer of marine natural compounds so far, molluscs, tunicates, coelenterates, echinoderms, and bryozoans [32,34]. In this ranking, the contribution of marine vertebrates, almost entirely represented by fish, is still rather modest (just right after coelenterates) [32,33], but certain factors, which will be commented on next, encourage further research into the potential antimicrobials that they may offer, especially from their skin mucus [36,37,38].

Fish are the oldest living vertebrates, with ancestors dating back to the mid-Cambrian period more than 500 million years ago. Among them, the ray-finned fishes (subclass *Actinopterygii*) are numerically dominant, with about 30,500 species, representing 95% of all fishes (about 32,000 species) and 50% of all vertebrates (about 60,000 species) (Table 1). They diverged about 385 My ago in the mid-Devonian period. The success of this divergence is represented by their enormous diversity as a consequence of having adapted to nearly any aquatic habitat since then [25,39]. This was made possible by a highly advantageous biological innovation (vertebrate evolution) and great genetic flexibility (gene duplication [40], teleost-specific whole-genome duplication events [41], and deletion of genome parts [42]) in a vast environment with extremely diverse conditions and high biological competition. However, this is also applicable to microorganisms.

Marine ecosystems are regulated by complex interactive fluxes that are primarily controlled by microorganisms due to the predominance of their biomass [43]. With a focus on viruses, bacteria, and fungi, some quantitative studies have estimated the abundance of each of the first two groups in the millions per milliliter of seawater [26,44,45]. The virus is the predominant microorganism in the ocean, accounting for about 10^30^ particles, about 15 times more than estimated bacteria (and archaea) [26]. There is little information on the quantitative abundance of fungi in aquatic environments, although it is assumed to be relatively high based on data on their enzymatic activity in certain environments compared to bacteria [46]. However, of note is not only their quantity, but also the extremely high diversity observed in all these groups, which also comprise pathogenic microorganisms [26,28,44,46].

As a result, fish have co-evolved under this selective pressure by also developing a complex network of defense mechanisms, such as the adaptive immune system [47,48,49]. However, although they have one of the earliest forms of adaptive immunity, their innate immunity still plays a central role in protecting them from and responding to infection [47,50], especially through a complex system of mucosal barriers responsible for fending off pathogens on first contact [47,50,51]. In fact, leukocyte distribution in fish is more organized in the mucosal tissues of the gut, gills, and skin than in the liver or gonads, for example [47,51]. Besides the cellular immune component, the humoral aspect of these tissues is of special relevance because of its antimicrobial function [52]. Among these major mucosa-associated lymphoid tissues (MALT), i.e., gut (GALT), gills (GIALT), and skin (SALT), mucosal glands are much more numerous in the skin [50,53], which is reasonable considering its continuous and intimate exposure to large amounts of microorganisms [26,44,45,46]. Together with the fact that the skin is the largest tissue of any organism and its mucus can be obtained non-invasively, the use of such fish fluid for biomedical purposes is very promising given its content of antimicrobial factors [54,55,56]. This is particularly true considering the large fish farming industry already established, which can valorize these previously overlooked natural by-products.

To explore the potential of fish mucus as a valuable source of antimicrobials, this review study conducted a search of the scientific databases PubMed, Scopus, and Web of Science. Keywords, such as “fish skin mucus,” in combination with “antimicrobial activity” or “composition,” were used in the search. The search period included studies published from the earliest available records to the present to ensure a thorough investigation. Inclusion criteria for the articles were: (i) published in English; (ii) in peer-reviewed journals; (iii) focused on the in vitro antimicrobial activity of fish skin mucus; and (iv) sufficiently described the mucus extraction method. Studies were selected based on their relevance to the topic and their adherence to predefined criteria.

## 3. Composition of Fish Skin Mucus in Innate Immunity Antimicrobial Molecules

The mucosal layer is a biochemical matrix that serves as a protective interface between the fish and the external environment [57]. Its multiple functions, which can be summarized as mechanical, physiological, and immunological, are dependent on its molecular components [37,57]. These are secreted by specific cell types (i.e., goblet, club, and sacciform cells) and the overall composition is influenced by developmental, hormonal, environmental, and nutritional factors [37,57,58]. The high-molecular-weight glycoproteins, called “mucins,” are the most representative component of the mucus and provide it with the characteristic gel structure that allows for the correct performance of all the above-mentioned functions [37,50,57,58,59]. The immunologic importance of this mucosal layer and its epithelial scaffold is demonstrated by the fact that its disruption increases the incidence and severity of infections [60,61]. In a recent example study, it was shown that such a disruption caused by the cyprinid herpesvirus 3 (CyHV-3) facilitates the occurrence of secondary infections that are ultimately responsible for exacerbated health complications and even death [61]. Besides the mucins themselves, which have been reported to have direct antimicrobial activity, fish skin mucus also contains a broad repertoire of antimicrobial factors apart from antibodies [36,50,51,58,62]. Mucus has often been recognized as a source of new antimicrobials [38,63,64]. Known compounds are summarized in this section.

### 3.1. Antimicrobial Peptides (AMPs)

AMPs, also known as Host Defense Peptides (HDPs), are gene-encoded peptides of up to approximately 80 amino acid residues, mostly characterized by a cationic, amphipathic chemical nature and antimicrobial properties. They are ancient innate immune molecules present in all groups of organisms. Their mature forms in eukaryotic cells are often cysteine-rich molecules with multiple intramolecular disulfide bridges. Through conservation or reduction of these bonds, some families of AMPs can modulate their type and/or level of activity [65,66,67,68]. For instance, in defensins (one of the most studied families of AMPs), some reports on a particular group of human beta-defensins indicate that the reduction of such bonds affects their function by disabling their chemotactic activities and triggering their direct antimicrobial ones [67]. Indeed, AMPs are generally known for their microbicidal activity exerted directly on the target microorganism, but they can also be endowed with potent immunomodulatory and receptor-mediated chemotactic activities, which together explain their broad antimicrobial activity against bacteria, protozoa, fungi, and both enveloped and non-enveloped viruses, as well as the difficulty of selecting resistant mutants against them [65,66]. In general, the mechanism of action for their direct antimicrobial activity is based on the affinity and fixation of the peptide to the generally anionic surface membrane of the pathogen and its subsequent destabilization through pore formation or, simply, bilayer disruption [65].

The large number of AMPs discovered (the Antimicrobial Peptide Database (APD) of the University of Nebraska Medical Centers currently counts 3569 and growing [accessed 5 May 2023]) can be classified into different families based on (i) their primary sequence; (ii) the presence and organization of various functional regions, such as the propeptide, which inhibits and protects the region corresponding to the mature peptide until its cleavage and is located at either the C- or N-terminal; (iii) their molecular structure, for which the simplest distinction is between linear and disulfide-stabilized peptides, and, in the latter case, the number, location, and association pattern of their cysteine residues; and (iv) the origin of the mature peptide, which may be produced directly after typical post-translational modifications and minor cleavages of accessory regions as it occurs in most cases, but may also be derived from the cleavage of a larger protein, often with a very different primary function, e.g., peptides derived from histone and ribosomal proteins [65,69,70,71].

Given the importance of these molecules in the innate immune system, they are extremely diverse in fish and include not only families of AMPs found in other animal groups, such as cathelicidins, defensins, hepcidins, and histone-derived peptides, but also exclusive fish AMP families, such as piscidins and pleurocidins [69,70]. Probably also for this reason, the skin mucus is the major source of AMPs in fish, with approximately 70% of all AMPs expressed in the skin compared to 52% and 29% expressed in the gills and the gut, respectively [50,53]. Besides expression, several AMPs were isolated from skin mucus, and their antimicrobial activities were tested. Representatives of several families of AMPs isolated from skin mucus are listed in Table 2.

Histone-derived AMPs were first described in fish [86] just a few years after their discovery in the Asian toad *Bufo bufo gargarizans* [87]. Robinette et al., (1998) [86] isolated two histone-like proteins (HLP-1 and HLP-2) in the epidermis of channel catfish that were found to be inhibitory to bacterial and fungal pathogens. Shortly thereafter, several histone-derived peptides were isolated from fish skin mucus [72,73,74,75]. In general, this family of AMPs is thought to be released from cells during infection-induced apoptosis [88]. Oncorhyncin III is a 66-residue N-terminal fragment of the non-histone chromosomal protein H6 from *O. mykiss* skin mucus and was shown to be active against gram-negative and gram-positive bacteria [80]. Other AMPs derived from larger proteins isolated from skin mucus are the peptidic fragments from the 40S and 60S ribosomal subunits. The AMP 40S ribosomal protein S30 was found in rainbow trout and showed antibacterial activity against gram-positive bacteria [85]. The 60S ribosomal proteins L40, L36A, and L35 were isolated from Atlantic cod (*G. morhua*) mucus extracts [76].

Other peptides found in fish skin mucus include myxinidin, pardaxin, pelteobagrin, and piscidin, all of which are unique to this group of animals and have been reported to have broad-spectrum antimicrobial activity. Myxinidin is a cationic 12-amino acid peptide isolated from the skin mucus of hagfish (*M. glutinosa*) [79]. Pardaxin was first isolated from the Red Sea Moses sole (*P. marmoratus*) and described as a single, helical, monomeric, acidic toxin [82]. Its antibacterial activity against gram-negative and gram-positive bacteria was subsequently demonstrated [89]. Pelteobagrin is a 20-amino acid amphipathic α-helical peptide and was identified in the skin mucus of yellow catfish (*P. fulvidraco*) [81]. The piscidin family also comprises α-helical peptides, with low molecular weight and cationic charge at physiological pH [90].

### 3.2. Proteins

Animal mucosa, in a broad sense, is characterized by the presence of mucins, which are glycosylated proteins responsible for providing viscoelastic and rheological properties, as well as trapping pathogens and contributing to cell surface signaling [36]. Additionally, they possess a diverse range of other proteins, including many with antimicrobial/immune-related and structural functions [50,51,57,58]. In this sense, a large number of defense proteins against pathogens have been described in fish mucosa, especially in the skin, besides the immunoglobulins IgG and the teleost-specific IgT [50,51,58].

Other types of glycoproteins have been found in fish skin mucus. For example, Ebran et al., (2000) [91] isolated and characterized glycoproteins from rainbow trout (*Oncorhynchus mykiss*), European eel (*Anguilla anguilla*), and tench (*Tinca tinca*) skin mucus. These proteins possess both α-helix and random coil structures and show antibacterial activity correlated with pore-forming properties. Transferrin glycoprotein has also been isolated from Atlantic cod [92] and Atlantic salmon (*Salmo salar*) [93] skin mucus. Transferrin is responsible for iron transporting in absorption, storage, and disposal sites in vertebrates. As all organisms require iron for their growth, transferrin plays an important role in the innate defense mechanisms of fish by binding to iron and reducing its availability to pathogens by chelating it [94].

Lectins are a diverse class of highly specific carbohydrate-binding proteins [95]. They have been found in the skin mucus of fish, where they provide an external defense mechanism via the agglutination process to stop pathogen penetration and colonization [96]. There are several types of different lectins depending on their structure; for example, C-type lectins, whose binding is dependent on Ca^2+^, F-type lectins or fucolectins, which are distinguished by their α-l-fucose recognition domain, galectin family or S-type, which require thiol, and pentraxins or pentameric lectins, or P-type lectins, which target glycoproteins containing mannose 6-phosphate [95]. The isolation of C-type lectins has been described in cichlid (*Symphysodon aequifasciata*) skin mucus [97]. Another example includes fucose-binding lectin (FBL, F-type lectin), which was identified in European sea bass (*Dicentrarchus labrax*) skin mucus [98] and is responsible for the agglutination, immobilization, and opsonization of microorganisms and phagocyte activation. Mannose-binding lectin (MBL, P-type lectin) and galectin were also found in Atlantic cod skin mucus [99]. MBL plays a significant role in opsonization and the initiation of the lectin pathway of complement activation, and galectin binds to pathogens and orchestrates several immune processes. Another type of MBL, named “pufflectin,” was reported in pufferfish (*Takifugu rubripes*) [100]. Pentraxins play an important role in inflammatory responses and pathogen recognition and have been identified in common skate (*Dipturus batis*) [101] and lumpsucker (*Cyclopterus lumpus*) [102] skin mucus. C-reactive protein (CRP) belongs to the pentraxin family, and it is part of the innate immune defense system because it has the ability to activate the classical complement pathway [103]. CRP was reported in tilapia (*Tilapia mossambica*) skin mucus, and its levels were found to increase in response to inflammation and necrosis [104].

Lysozyme (N-acetylmuramide glucanohydrolase or muramidase) is a bacteriolytic enzyme and an important component of the immune system. It has been reported in the skin mucus of several fish species, including mrigal carp (*Cirrhinus mrigala*), catla (*Catla catla*), spotted snakehead (*Channa punctata*), Japanese eel (*Anguilla japonica*), and Nile tilapia (*Oreochromis niloticus*) [105,106,107]. Given its ability to hydrolyze the bond between N-acetylmuramic acid and 3-acetyl amino-2-deoxy-D-glucose residues of the mucopolysaccharide found in bacterial cell walls [108], it acts directly on gram-positive bacteria. In gram-negative bacteria, lysozyme can also attack the inner peptidoglycan layer after the disruption of the outer wall by complement and other enzymes [106].

Proteases are enzymes of great importance in the mechanisms of the immune system. Their role is to hydrolyze the peptide bonds of proteins. Proteases can be classified into serine, cysteine, aspartic, and metalloproteases based on their catalytic mechanisms [58]. They are associated with resistance to infection because of their ability to degrade the proteins of pathogens. Proteases, including trypsin (serine protease), cathepsin B and L (cysteine proteases), cathepsin D (aspartic protease), aminopeptidases, and metalloproteases, have been reported in the skin mucus of several species, such as rainbow trout [109], Japanese eel [110], European eel [111], catfish (*Parasilurus asotus*) [75], and Atlantic salmon [112].

Cytoskeletal proteins with potential antimicrobial activity have also been reported in fish skin mucus. For example, keratin has been identified in skin mucus from lumpsucker [102], Atlantic cod [99], European sea bass [98], and gilthead sea bream (*Sparus aurata*) [113]. Although keratin is a structural protein, pore-forming properties have also been described in keratin from the skin mucus of rainbow trout [114] and may therefore contribute to host defense against water-borne pathogens. Likewise, actin is a structural protein involved in several roles associated with cellular membranes, such as cell migration, phagocytosis, pinocytosis, cytokinesis, and cytoplasmic streaming [115]. Beta actin has been reported in lumpsucker [102], European sea bass [98], Atlantic cod [99], and gilthead sea bream [113,116]. Increased actin fragmentation by proteases has been linked to stress situations, and actin fragments generated could trigger an immune response [117].

### 3.3. Other Components

**Figure 1:**
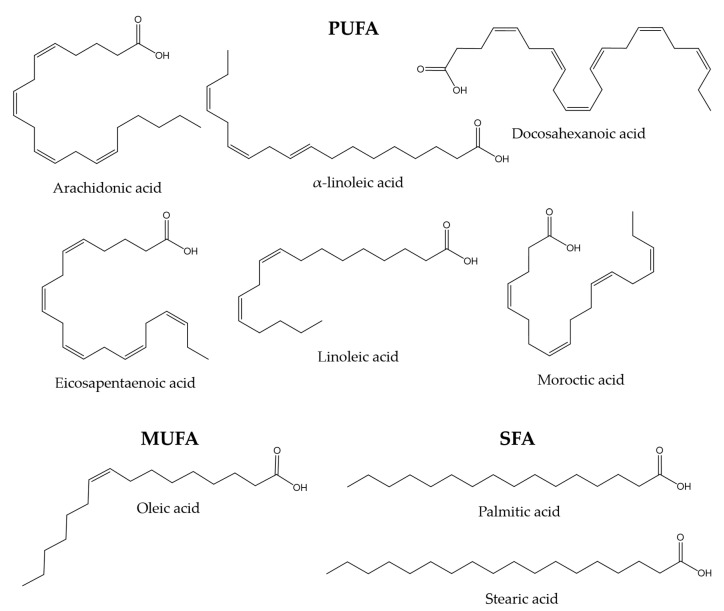
Chemical structures of some MUFA, PUFA, and SFA molecules found in the skin mucus of gilthead sea bream and flathead grey mullet [118,119].

The lipid composition of fish skin mucus has not been studied as thoroughly as other mucous secretions, such as gut mucus. However, some studies show that skin mucus may also be a significant source of lipids. Mono-unsaturated fatty acids (MUFA), such as oleic acid, poly-unsaturated fatty acids (PUFA), such as linoleic, alpha-linoleic, docosahexaenoic, arachidonic, eicosapentaenoic, and moroctic acid, and saturated fatty acids (SFA), such as palmitic and stearic acid, have been reported in gilthead sea bream and flathead grey mullet (*Mugil cephalus*) [118,119]. Lipids are thought to be involved in maintaining the internal structure of the mucus through interactions with glycoproteins [120]. Figure 1 shows the chemical structures of the compounds mentioned in this paragraph.

**Figure 2:**
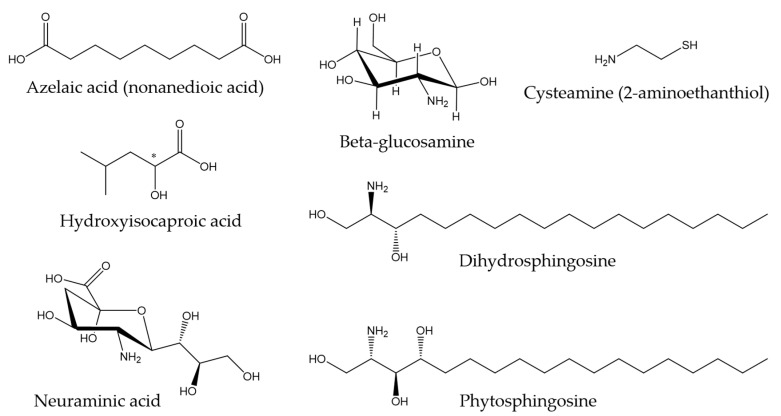
Chemical structures of other metabolites found in skin mucus of fathead minnow and pool barb [121,124].

Regarding other types of molecules, Ekman et al. (2015) [121] described the metabolite profile of fathead minnow (*Pimephales promelas*) skin mucus with the aim of providing a tool for environmental monitoring and surveillance. Some of the metabolites found are associated with antibacterial properties. For instance, azelaic acid has been shown to inhibit bacterial growth by interfering with protein synthesis [122], and hydroxyisocaproic acid has been found to be effective against both bacteria and fungi [123]. In another study conducted by Patel et al., (2020) [124], the metabolic profile of the skin mucus of the pool barb (*Puntius sophore)* was characterized. In this research, compounds found with proven antimicrobial activity included amino sugars, such as glucosamine and neuraminic acid, cysteamine (organic disulfide), dihydrosphingosine (amino alcohol), and phytosphingosine (sphingolipid), among others. Figure 2 shows the chemical structures of the compounds mentioned in this paragraph.

## 4. Antimicrobial Activity of Fish Skin Mucus

**Figure 3:**
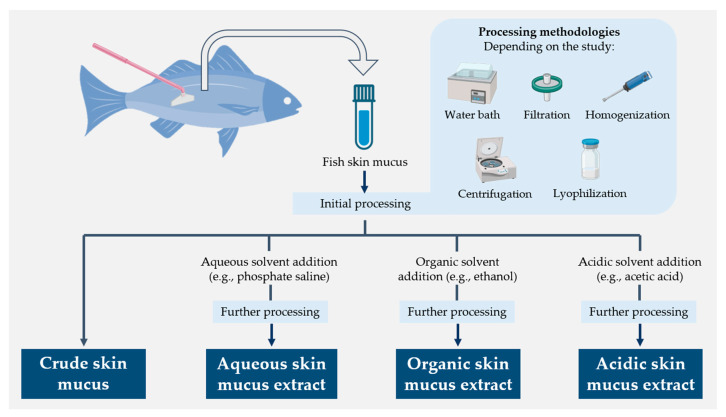
Schematic diagram summarizing the different molecular extraction approaches used for fish skin mucus samples [63,125].

The humoral component of fish skin mucus has been extensively studied for its high content in molecules endowed with antimicrobial properties and, thus, its potential for implementation in biomedical and veterinary applications [38,50,51,58]. Such a variety of compounds has also necessitated the use of different molecular extraction approaches in mucus samples (Figure 3) [63,125]. However, it appears that the amount of functional data is relatively small compared to the overall progress made in researching and developing new methods. This section summarizes the activity demonstrated against different groups of microorganisms by different types of fish skin mucus extracts, mentioning the techniques used to determine this activity when specified in the studies.

### 4.1. Antibacterial Activity of Fish Skin Mucus Extracts

Fish skin mucus has proven to be effective against bacteria that affect not only fish, but humans as well. Table 3 summarizes the results of an extended list of studies on antibacterial activity of fish skin mucus extracts. In total, there are 47 fish species represented, most of which are teleosts (exceptions include *Myxine glutinosa* (Myxini) and *Dasyatis pastinaca* (Elasmobranch)). The different extraction methods can be divided almost entirely into aqueous, organic, and acidic extractions. Some authors also used crude mucus with only minor processing steps.

#### 4.1.1. Aqueous Extractions

Of all the experiments presented in Table 3, the most frequently used extraction method was the aqueous one. The most commonly employed solvents in these studies, listed in order of frequency of use, were physiological saline, water, ammonium bicarbonate, and Tris-buffered saline. Further information about these studies can be found in Table S1.

Although aqueous extraction was the most popular extraction method, it also showed the least antibacterial activity. This was particularly evident in those experiments where different extraction methods were compared. In some experiments, aqueous extracts did not show any antibacterial activity [127,137,138,139,140,141,151]. For example, Subramanian et al., (2008) [140] found antimicrobial agents, such as lysozyme, cathepsin B, and trypsin-like proteases, in the aqueous skin mucus extracts of several fish species, but they did not exert any antimicrobial activity. Al-Rashed et al., (2018) [127] used aqueous and acidic extracts of the skin mucus of the climbing perch (*Anabas testudineus*), but only found antibacterial activity in the latter. Similarly, Hellio et al., (2002) [138] performed aqueous and organic extractions of the skin mucus of the ballan wrasse (*L. bergylta*), but only observed antibacterial activity with the organic extracts. Subhashini et al., (2013) [151] did not find antimicrobial activity in the aqueous extract of the skin mucus of tinfoil barb fish (*Barbonymus Schwanenfeldii*), even if the amount of protein was higher than in the organic extracts obtained in parallel.

The lack of activity of these extracts has been attributed by different authors to several causes: (i) inactivation of enzymes, such as lysozyme, trypsin, or proteases, by the high incubation temperatures and/or low pH conditions used in the procedure [140]; (ii) low concentration of antimicrobial compounds in the media, probably because some of these enzymes regulate their production [161]; and (iii) inter- and intraspecies variability of mucus composition, influenced by both internal (e.g., sex and developmental stage) and external factors (e.g., stress, hyperosmolarity, pH, and infection) [62]. This last point is particularly noticeable in the species studied in several studies performing aqueous extractions. The aqueous skin mucus extract of the common snakehead (*Channa striata*) has been shown in some studies to be active against gram-negative and gram-positive bacteria [130,132,133]. However, Wei et al., (2010) [131] also tested the antibacterial activity of this extract and found inhibition only against *Aeromonas hydrophila* but not against other bacteria previously tested (i.e., *Bacillus subtilis*, *Klebsiella pneumoniae*, *Proteus vulgaris,* and *Pseudomonas aeruginosa*).

In other studies, the aqueous skin mucus extract did inhibit both gram-negative and gram-positive bacteria [130,132,133,134,135,149,152,153,155,156,157]. Guardiola et al., (2014) [134] demonstrated that an aqueous extract of the skin mucus of grouper (*Epinephelus marginatus)* inhibited gram-positive and gram-negative bacteria. They also found high levels of lysozyme, alkaline phosphatase, esterase, protease, and antiprotease activities, which are associated with defense against bacterial infections. In a study by Kumari et al., (2019), [156] the aqueous skin mucus extract of several carp species exhibited high antibacterial activity against both gram-negative and gram-positive bacteria.

#### 4.1.2. Organic Extractions

For the organic extractions, the most used solvents have been ethanol and dichloromethane. Some authors performed alcoholic extractions and then partitioned distilled water with dichloromethane to obtain aqueous and organic phases [138,151]. High antibacterial activity has been reported for organic fish skin mucus extracts (see Table 3 and Table S2 for further details). Indeed, in some studies, all bacteria tested were inhibited, both gram-positive and gram-negative [76,138,145,151]. The main reasons explaining such activity are that (i) the presence of hydrophobic groups is often a common feature of antimicrobial molecules because of their affinity for membranes and their ability to disrupt them [162]; and (ii) these extracts are enriched in hydrophobic molecules because organic solvents favor their isolation by reducing the interactions between hydrophobic groups, which hinders their aggregation [163]. In fact, Mahadevan et al., (2019) [145] obtained greater inhibitory activity against gram-negative and gram-positive bacteria using organic mucus extracts compared to aqueous ones. Hellio et al., (2002) [138] correlated high antimicrobial activity with low polarity of the solvents used; they also showed that extracts from the dichloromethane phase were more active than those from the aqueous phase. In a study by Bergsson et al., (2005) [76], an organic (acetonitrile (ACN) + 1% trifluoroacetic acid (TFA)) extract of cod skin mucus exhibited high antimicrobial activity against gram-positive and gram-negative bacteria. In these extracts, they also identified four peptides with known antimicrobial activity, i.e., those derived from the histone H2B and the 60S ribosomal proteins L40, L36A, and L35.

In some studies, the same species were used to obtain the organic extracts of skin mucus. García-Marciano et al., (2019) [141] and Wibowo and Maftuch (2015) [143] tested the organic skin mucus of tilapia against *V. harveyi,* and in both studies, bacterial growth was inhibited. Katra et al., (2016) [139] and Hellio et al., (2002) [138] employed the same methodology to obtain organic skin mucus extracts from ballan wrasse (*L. bergylta*) and these two studies, no activity against gram-positive bacteria was found. However, Hellio et al., (2002) [138] found antimicrobial activity against gram-negatives, while Katra et al., (2016) [139] did not. These contradictory results were attributed to the possible development of antimicrobial resistance in the strains used, or to seasonal, housing, or dietary differences between the two studies that may have affected the experimental animals.

#### 4.1.3. Acidic Extractions

For acidic extractions, the most common solvent was acetic acid (AA) followed by TFA. In general, acidic extracts showed greater antibacterial activity than other extracts (see Table 3 and Table S3 for further information) [124,127,131,137,140,141,142,150,151]. In most studies using acidic extractions to determine antibacterial capacity, all bacteria tested were inhibited [124,131,137,140,142,154]. This may be due to the presence of cationic peptides and defensive low-molecular-weight proteins. This type of molecule has been shown to be more soluble in mildly acidic solutions [164].

Subramanian et al., (2008) [140] tested the antimicrobial activity of brook trout (*Salvelinus fontinalis*), haddock (*Melanogrammus aeglefinus*), and hagfish acidic (AA) skin mucus extracts against gram-negative (*A. salmonicida*, *E. coli*, *Listonella anguillarum*, *Salmonella enterica*, *P. aeruginosa*, *Yersinia ruckeri*) and gram-positive (*Staphylococcus epidermidis*) bacteria, and all species studied were inhibited. Hagfish mucus was the most active one, and its protein profile showed mainly low-molecular-weight proteins below 20 kDa. This was related to the fact that hagfish are evolutionarily the most primitive of the species studied and they lack essential components of adaptive defense, as well as their presence in muddy ocean bottoms, which requires a greater amount of antimicrobial components for their survival [140].

Nigam et al., (2017) [154] identified the antimicrobial protein histone H2B in acidic (AA and TFA) mrigal carp skin mucus extracts. However, the TFA extract only inhibited two (*Salmonella paratyphi*, *Vibrio cholerae*) of the five bacteria tested. Patel et al., (2020) [124] detected some metabolites in the acidic (AA) pool of barb mucus extract that were associated with antimicrobial activity, such as 10-nitro-9Z,12Z-octadecadienoic acid, 3alpha,6beta,7alpha-trihydroxy-5betacholan-24-oic acid, 1-octanoyl-rac-glycerol, dihydrosphingosine, phytosphingosine, 5beta-chol-2-en-24-oic acid, neuraminic acid, glucosamine, and cysteamine. It has been described that antimicrobial lipids probably act by inducing cell wall and membrane destabilization of bacteria [165].

#### 4.1.4. Crude Mucus

Finally, some studies have evaluated the activity of fish skin mucus in its almost raw form, without any type of solvent extraction. Table 3 and Table S4 provide additional information about these studies. Sanahuja et al., (2019) [128] compared the crude skin mucus of gilthead sea bream, European sea bass, and meagre (*Argyrosomus regius*). In particular, meagre mucus showed biocidal activity against all bacterial species tested, i.e., *E. coli*, *V. anguillarum,* and *P. anguilliseptica* (all gram-negative), which was associated with higher levels of non-specific defenses, such as protease and carboxylesterase activities. Fuochi et al., (2017) [158] studied the antibacterial activity of common stingray (*D. pastinaca*) crude skin mucus and found that it inhibits the bacterial growth of gram-negative, but not gram-positive, bacteria. This observation was attributed to a strong interaction between the outer membrane (present in gram-negatives only) and the biomolecules present in the mucus. They also demonstrated the presence of chitinase 1, an enzyme involved in the degradation of chitin [158].

Other authors have compared the antibacterial activity between the crude mucus and some of its solvent extracts. Kumari et al., (2019) [156] found that the crude mucus of several carp species shows higher antibacterial activity than its aqueous extract. Wei et al., (2010) [131] compared crude, aqueous, and acidic mucus from common snakehead. Although the crude mucus was found to contain a higher amount of protein than the other extracts, it inhibited only the fish pathogen *A. hydrophila*. In this work, the crude mucus did not exert any effect against the human bacteria pathogens tested, unlike the other extracts.

### 4.2. Antifungal Activity of Fish Skin Mucus Extracts

Fish skin mucus has also shown antimicrobial activity against fungal pathogens. However, the number of studies evaluating antifungal activity is much lower than the number of studies evaluating antibacterial activity. Of the 39 selected studies that evaluated antibacterial and antifungal activity, 28 evaluated only antibacterial activity, two evaluated antifungal activity exclusively, and nine evaluated both. The antifungal studies conducted using fish skin mucus are shown in Table 4.

Several studies have produced mixed results using crude fish skin mucus. On the one hand, the antifungal activity of crude skin mucus of catla, mrigal carp, and European eel inhibited the growth of *Aspergillus awamori*, *Colletotrichum falcatum,* and *Fusarium oxysporum* in the study by Pethkar et al., (2017) [146]. Fuochi et al., (2017) [158] also found that the crude skin mucus of the common stingray was active against *Candida albicans*, *Candida glabrata,* and *Candida tropicalis*. On the other hand, Hisar et al., (2014) [159] tested the crude skin mucus of rainbow trout against *C. albicans* and *Candida parapsilosis*, but no antifungal activity was observed. Ikram et al., (2013) [167] screened the antifungal activity of crude and aqueous (i.e., PBS and water) skin mucus of Asian swamp eel (*Monopterus albus*) against *C. albicans*, *Candida krusei*, *Cryptococcus neoformans,* and *Fusarium* spp., but only the water extract revealed an inhibitory effect, with activity against all the fungi tested and mostly against *Fusarium* spp.

A few studies only investigated aqueous mucus extractions. For example, walking catfish (*Clarias batrachus*) aqueous skin mucus inhibited *Aspergillus niger*, *Aspergillus nidulans*, *Fusarium moniliforme*, *C. albicans,* and *Trichoderma koningi* in the study by Loganathan et al., (2011) [149]. Balasubramanian et al., (2011) [153] tested the antifungal activity of catla, silver carp (*Hypophthalmichthys molitrix)*, rohu *(Labeo rohita),* and grass carp *(Ctenopharyngodon idella)* aqueous skin mucus against *Aspergillus flavus*, *A. niger*, *C. albicans*, *Mucor globosus,* and *Rhizopus arrhizus*. The catla and rohu extracts inhibited all the species tested, the silver carp extract inhibited *M. globosus*, *A. flavus,* and *A. niger*, and the grass carp extract inhibited all species except *A. niger*.

Other studies carried out aqueous and organic or acidic extractions in parallel and compared their activity. Hellio et al., (2002) [138] screened the antifungal activity of the aqueous and organic skin mucus extracts of pollock (*Pollachius virens*), ballan wrasse, European flounder (*Platichthys flesus)*, common sole (*Solea solea*), and brill (*Scophthalmus rhombus*) against *Candida brusei*, *C. albicans*, *C. tropicalis*, *Saccharomyces cerevisiae,* and *Issatchenkia orientalis*. Antifungal activity was found only in the aqueous and organic fractions of the organic extraction of brill mucus against *C. tropicalis, I. orientalis,* and *S. cerevisiae*. In the study by Subramanian et al., (2008) [140], the aqueous, acidic, and organic skin mucus extracts of arctic char (*Salvelinus alpinus)*, brook trout (*Salvelinus fontinalis)*, Eurasian carp (*Cyprinus carpio* sub sp. Koi), striped bass (*Morone saxatilis)*, haddock (*Melanogrammus aeglefinus),* and hagfish were tested against *C. albicans*. The results showed that the acidic skin mucus of arctic char, brook trout, and hagfish exerted fungicidal activity, and the acidic skin mucus extracts of European carp and striped bass were fungistatic. Mahadevan et al., (2019) [145] showed that the aqueous and organic skin mucus extracts of the giant mudskipper (*Periophthalmodon schlosseri)* inhibited *C. albicans*, *A. flavus*, *Mucor* sp., and *Trichoderma longibriachtin*.

Bergsson et al. (2005) [76] extracted the skin mucus of Atlantic cod using 60% ACN containing 1% TFA. They evaluated its activity against *C. albicans*, which was fully eliminated when Medium E (salt solution) was added to the medium. Without Medium E, only mild inhibition was observed. In another study, al-Arifa et al., (2011) [166] demonstrated that the use of alkali treatments to induce the production of skin mucus in rohu before its collection inactivated its antifungal properties, and they were not restored after the neutralization of its pH. Instead, it favored the growth of *C. albicans*.

The antifungal activity of mucus components, such as lysozyme [168,169] or chitinases [170], have been demonstrated. Some studies suggest that the lysozyme may disrupt cell wall integrity by hydrolyzing the glycosidic linkages between cell wall proteins and polysaccharides [168]. Other hypotheses include that lysozyme is likely to induce apoptosis in this fungus accompanied by activation of membrane potassium channels [169]. Regarding chitinases, they catalyze the hydrolysis of chitin, a main component of the cell wall in fungi [170].

### 4.3. Antiviral Activity of Fish Skin Mucus

Information on the antiviral activity of fish skin mucus extracts is scarce to date. Raj et al., (2011) [171] investigated the role of carp epidermal mucus as an innate immune barrier against CyHV-3 entry. They found that skin mucus inhibits CyHV-3 binding on epidermal cells and leads to a significant reduction in the number of viral plaques. This reduction only occurred when cells were pre-incubated with mucus, but not when mucus was added after the incubation period. Most of the studies, however, report antiviral activity of compounds that had been previously isolated from skin mucus. For instance, Valero et al., (2020) [172] reported the presence of NK-lysin in Atlantic salmon skin mucus, and Falco et al., (2019) [173] demonstrated its antiviral activity against spring viremia of carp virus (SVCV) by inhibiting not only the binding of viral particles to host cells, but also the fusion of virus and cell membranes. Beta-defensins, an important factor in the antimicrobial barrier function of the skin [174], have also been shown to have antiviral activity against another rhabdovirus, the viral haemorrhagic septicaemia virus (VHSV) [175]. Furthermore, lysozyme [176] and piscidin (piscidin 1) [177] have antiviral activity. However, the scarcity of systemic and functional data shows that more research on the antiviral role of fish mucus is required to draw clear conclusions and to develop new strategies to treat viral infections.

## 5. Omics Techniques as a Promising Tool in Fish Skin Mucus Research

The field of fish skin mucus research has generally been limited by the complexity of its composition, as well as the interactions between its components. Omic techniques have recently emerged to provide a holistic approach to cellular components and their interactions, providing an effective tool towards a deeper understanding of marine systems [178]. The progress of these techniques has allowed the field of studies of fish skin mucus to grow quickly in recent years [36]. These techniques have been applied to fish skin mucus research in different topics, such as welfare, health, and nutrition. However, the discovery of antimicrobial agents through these methods is an underexplored opportunity.

Genomics have provided insights into molecular and genetic mechanisms in fish. Ao et al., (2015) [179] sequenced and assembled the genome of *Larimichthys crocea* using a bacterial artificial chromosome and a whole-genome shotgun hierarchical strategy. They identified 159 genes related to mucin biosynthesis and mucus production based on previous studies in mammals, thus suggesting that the mucin synthetic pathway is conserved between fish and mammals. Carda-Diéguez et al., (2017) [180] searched for genomic and metagenomic evidence in wild eel to discover if fish mucus could constitute an adequate niche for the evolution of mucosal aquatic pathogens in natural environments. The results obtained suggest that skin mucus concentrates in bacteria present in water with abilities to attach, resist innate immunity, and compete with other bacteria, and that it favors the exchange of genes encoding these functions.

Transcriptomics provides information on the RNA transcripts produced by the genome, from protein coding (mRNA) to noncoding RNA. A recent study examined the efficacy of whole-transcriptomic profiling of mahi-mahi epidermal mucus as a method for oil exposure detection using RNASeq [181]. Transcripts involved in immune response, cardiotoxicity, and calcium homeostasis showed differential expression after oil exposure, which indicates that mucus is a promising source for noninvasive monitoring techniques. Parida et al., (2018) [182] examined the transcriptome of immune-relevant genes in the mucus of infected rohu to characterize mucosal immune responses. The results show, in general, the upregulation of immune-related transcripts, such as interleukins, toll-like receptor 22, and lysozyme G, which broadens our knowledge of mucosa-associated molecular events that occur during infections and reinforces the role of mucus as the first line of defense against pathogens.

Proteomics is the characterization of proteins expressed in an organism. It is the most studied omic technique in fish mucus research. Proteomics provides information about the entire effect of the gene expression process and encompasses post-transcriptional and post-translational protein expression regulation [183]. Proteomic profiles of the skin mucus of gilthead seabream [113], Atlantic cod [99], Atlantic salmon [184], mudskipper [185], discus fish (*Symphysodon* spp.) [186], European sea bass [98], and lumpsucker [102] have been published, allowing for the possibility of comparative studies to better understand the dynamics of fish mucus. Moreover, the proteomic profile of fish mucus subjected to different types of stress has been studied, such as chronic wounds [116], bacterial infection [187,188], parasitic infection [189], artificial stressors [190], and sample collection [191]. These types of studies expand our knowledge of proteomic changes associated with immune processes, and they can be a starting point to develop a powerful tool to identify bioindicators of fish welfare and physiological status via non-invasive methods.

Metabolomics can be defined as the quantitative complement of all low-molecular-weight molecules present in cells in a particular physiological or developmental state [192]. Metabolomics is situated downstream of proteomics, transcriptomics, and genomics, thus making metabolomics extremely useful for understanding organism responses and for biomarker discovery [37]. Analytical methods in metabolomics commonly include mass spectrometry (MS), often in conjunction with gas chromatography (GC) and liquid chromatography (LC), and nuclear magnetic resonance (NMR). Studies on fish metabolomics cover a wide range of fields of knowledge, including fish physiology and development, pollutants’ effects on fish, fish condition and disease, and fish as foodstuff [193]. Ekman et al., (2015) [121] characterized fathead minnow skin mucus metabolites using LC-MS in order to report a minimally invasive sampling method. Results indicate that the metabolome varies between sexes and that it is very sensitive to chemical exposure, suggesting that metabolomics is useful for environmental monitoring and surveillance. Wen et al., (2020) [194] analyzed discus fish skin mucus metabolites using LC-MS/MS-based metabolomics and found changes in metabolite profiles as they entered the stage of parental care; those variations were sex-specific in parental fish. Ivanova et al., (2018) [195] employed LC-MS metabolomics to detect small metabolites using different sample collection techniques. The results suggest that the scraping method to collect mucus is more invasive than other methods due to changes in metabolic profiles.

A combination of different omic techniques pursues the integration of different biological entities to understand their interrelation and the functioning of larger systems, and serves to identify new biomarkers in specific tissues. For instance, multiomic analysis of gilthead sea bream skin mucosa was carried out by measuring the entirety of biomolecules differentially expressed by means of skin transcriptomic analyses and the modification in mucus layer exudation by the analysis of the mucus proteome [196]. Information on fish mucus at genomic, transcriptional, protein, and metabolic levels has significantly moved fish mucus research forward. Applications associated with fish health and welfare, monitoring, food safety, and aquaculture production can be achieved using omic techniques. Important insights can be found not only within those techniques but also through understanding the interactions between them.

## 6. Conclusions

Besides being a key component in several physiological functions, fish skin mucus provides an effective chemical and physical barrier against pathogens. The performance of this activity is highly dependent on mucus composition. Therefore, the choice of a suitable molecular extraction method is crucial for its antimicrobial use in other applications. Indeed, notable differences in antimicrobial activity have been shown for the different types of extracts reviewed here, which is particularly relevant in those studies comparing different extraction methods on the same samples. In general, acidic extracts, followed by organic ones, showed the highest antimicrobial activity. This may be because these procedures favor the isolation of cationic and/or amphipathic antimicrobial compounds, such as AMPs, their enrichment in the final extracts and, apparently, the minimization of molecular inactivation events.

The general analysis of the studies reviewed here shows that 76% of the authors tested antibacterial activity against both gram-negative and gram-positive bacteria, while the remaining 24% used gram-negative bacteria only. In this sense, *E. coli* and *S. aureus* were the most commonly used gram-negative and gram-positive bacteria, respectively. With regard to the origin (or host target) of the pathogens tested, most studies used both human and fish pathogens (57%), while 35% and 8% used only human and fish pathogens, respectively. In some experiments, the (mostly acidic and organic) extracts inhibited the replication of gram-negative, but not gram-positive, bacteria. One of the reasons for this may be the higher affinity of the cationic antimicrobial molecules in the extracts for the negatively charged outer membrane of gram-negative bacteria, which is not present in gram-positive bacteria.

Focusing now on the procedures to quantify antimicrobial activity, and, mostly, antibacterial and antifungal activity, the most common methods employed are, in order, disc diffusion (46%), agar-well diffusion (26%), broth dilution (12%), optical density measurement (7%), and broth dilution recorded by optical measurement (5%). The disc diffusion and agar-well diffusion assays are simple, inexpensive, and intuitive to interpret; however, their low sensitivity makes them unsuitable for comparative studies and for determining precise inhibitory factors, such as the minimum and half-maximal inhibitory concentration (MIC and IC50, respectively). Therefore, and as a preview of the next section, it would be recommended that the methods used to evaluate the antimicrobial activity of compounds in this particular area of research be standardized to allow for appropriate comparisons.

## 7. Recommendations and Future Perspectives

This review emphasizes the importance of mucus composition for its antimicrobial activity. However, this composition may differ notably depending on several factors, such as species, sex, age, and environment. Therefore, more information on these factors should be included in such studies to improve reproducibility. A practical option could be to focus these studies on animals at harvesting stages in order to normalize the results at the most appropriate time for industrial exploitation. In this sense, it would also be of great interest to develop efficient technologies for the collection of fish skin mucus at harvesting sites.

However, the momentum needed to accelerate progress in these lines of research and their translation into practice requires a substantial increase in the need for these products. The urgency for new antimicrobials noted in the introduction to this review is one such need. However, new ideas that define the targets against which these compounds could already make a difference would greatly accelerate their development. Examples include the use of marine antimicrobials in high ionic strength environments, such as the mucosal tissues of cystic fibrosis patients [197] or food preservation [198]. In this line of research, it would also be interesting to study formats to increase their stability and to improve their delivery; for example, by encapsulating them in micro- or even nanomaterials.

Finally, it is important to reiterate that the study and understanding of the fish skin mucus interactome using omic techniques provides new, unprecedented opportunities for antimicrobial drug discovery. Multiomics may also allow for the discovery of clinically important metabolites, interactions between components, and the mechanisms by which components exert their antimicrobial activity.

## Figures and Tables

**Table 1:** Estimates of number, mass, and time of origin on Earth for taxa relevant to this study.

Variable ^1^	Virus	Bacteria	Fungi	Human	Livestock	Fish
Number [19]	10^31^	10^30^	10^27^	10^10^	10^10^	10^15^
Mass, Gt C [19]	0.2	70	12	0.06	0.1	0.7
Time, years	3.8 × 10^9^ [20] ^2^	3.8 × 10^9^ [21]	1.5 × 10^9^ [22]	3 × 10^5^ [23]	84 × 10^6^ [24]	5 × 10^8^ [25]

^1^ This table is intended to give a general idea of the enormous differences in magnitude in terms of number, mass, and time since emergence of the various taxa relevant to this study, in order to understand the great challenge addressed in this study. Of course, not all microorganisms pose a pathogenic threat. In fact, most of the antibiotics used come from bacteria and fungi [16], most of the viruses are bacteriophages that modulate the density of bacterial communities in the oceans [12,26], and, as a whole, microorganisms are critical elements in the regulation of ecosystems [12,21,26,27]. ^2^ Like other studies, the estimated time of the origin of viruses on Earth is proposed here to be the same as that of the origin of life.

**Table 2:** Examples of AMPs isolated from skin mucus.

Family	AMP	Species	Ref.
Histone 2A *	N-acetylated Histone 2A	*Oncorhynchus mykiss*	[72]
	Hipposin	*Hippoglossus hippoglossus*	[73]
	Parasin I	*Parasilurus asotus*	[74,75]
Histone 2B *	Histone H2B	*Gadus morhua*	[76]
Histone H1 *	Oncorhyncin II	*O. mykiss*	[77]
	SAMP H1	*Salmo salar*	[78]
Myxinidin	Myxinidin	*Myxine glutinosa*	[79]
Non-histone chromosomal protein H6 *	Oncorhyncin III	*O. mykiss*	[80]
Pelteobagrin	Pelteobagrin	*Pelteobagrus fulvidraco*	[81]
Pardaxin	Pardaxin I, II	*Pardachirus marmoratus*	[82]
Piscidin	Piscidin 1, 2, 2β	*G. morhua*	[83]
Pleurocidin	Pleurocidin	*Pleuronectes americanus*	[84]
Ribosomal proteins *	40S Ribosomal protein S30	*O. mykiss*	[85]
	60S Ribosomal protein L35, L36A, L40	*G. morhua*	[76]

* peptides derived from these proteins.

**Table 3:** Antibacterial effect observed in extractions of mucus of different fish species.

Extraction	Aqueous								Organic				Acidic				Crude		Ref.
Solvent	W		AB		PS		TBS		ET		DCM		AA		TFA				
Gram	+	−	+	−	+	−	+	−	+	−	+	−	+	−	+	−	+	−	
Perciformes order																			
*Amphiprion clarkii*																			[126]
*Anabas testudineus*																			[127]
*Argyrosomus regius*																			[128]
*Channa argus*																			[129]
*Channa marulius*																			[130]
*Channa micropeltes*																			[130]
*Channa striata*																			[130,131,132,133]
*Dentex dentex*																			[134]
*Dicentrarchus labrax*																			[128,134,135,136]
*Epinephelus marginatus*																			[134]
*Epinephelus tauvina*																			[137]
*Labrus bergylta*																			[138,139]
*Morone saxatilis*																			[140]
*Oreochromis niloticus*																			[141,142,143]
*O. mossambicus*																			[144]
*Pagellus bogaraveo*																			[135]
*P. schlosseri*																			[145]
*Sparus aurata*																			[128,134,136]
*Umbrina cirrosa*																			[134]
Anguilliformes order																			
*Anguilla anguilla*																			[135,146]
Siluriformes order																			
*Arius maculatus*																			[147]
*Clarias batrachus*																			[142,144,148,149]
*Heteropneustes fossilis*																			[133]
*Rita rita*																			[150]
Cypriniformes order																			
*B. schwanenfeldii*																			[151]
*Catla catla*																			[146,152,153]
*Cirrhinus mrigala*																			[146,154,155]
*Ctenopharyngodon idella*																			[152,153,156]
*Cyprinus carpio*																			[140,156]
*H. nobilis*																			[152,153,156,157]
*Labeo rohita*																			[152,153]
*Puntius sophore*																			[124]
Anabantiformes order																			
*Channa gachua*																			[130]
*Channa punctatus*																			[130,150,155]
Myliobatiformes order																			
*Dasyatis pastinaca*																			[158]
Gadiformes order																			
*Gadus morhua*																			[76,138]
*M. aeglefinus*																			[140]
*Pollachius virens*																			[138]
Myxiniformes order																			
*Myxine glutinosa*																			[140]
Salmoniformes order																			
*Oncorhynchus mykiss*																			[159]
*Salvelinus alpinus*																			[140]
*Salvelinus fontinalis*																			[140]
Pleuronectiformes order																			
*Platichthys flesus*																			[138]
*Scophthalmus rhombus*																			[138]
*Scophthalmus maximus*																			[136]
*Solea senegalensis*																			[160]
*Solea solea*																			[138]

Antibacterial effect: 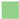 no effect; 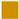 low or variable inhibition; 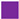 strong inhibition. W: water; AB: ammonium bicarbonate; PS: phosphate saline; TBS: Tris-buffered saline; ET: ethanol; DCM: dichloromethane; AA: acetic acid; TFA: trifluoroacetic acid. Fish family and species full names: *Anabantidae*: *Anabas testudineus* Bloch; *Anguillidae*: *Anguilla anguilla* L.; *Ariidae*: *Arius maculatus* Thunberg; *Bagridae*: *Rita rita* Hamilton; *Channidae*: *Channa argus* Cantor, *Channa gachua* Hamilton, *Channa marulius* Hamilton, *Channa micropeltes* G. Cuvier, *Channa punctatus* Bloch, *Channa striata* Bloch; *Cichlidae*: *Oreochromis mossambicus* W. K. H. Peters, *Oreochromis niloticus* L., *Clarias batrachus* L.; *Cyprinidae*: *Barbonymus schwanenfeldii* Bleeker, *Catla catla* Hamilton, *Cirrhinus mrigala* Hamilton, *Ctenopharyngodon idella* Valenciennes, *Cyprinus carpio* L., *Hypophthalmichthys nobilis* Richardson, *Labeo rohita* Hamilton, *Puntius sophore* Hamilton; *Dasyatidae*: *Dasyatis pastinaca* L.; *Gadidae*: *Gadus morhua* L., *Melanogrammus aeglefinus* L., *Pollachius virens* L.; *Heteropneustidae*: *Heteropneustes fossilis* Bloch; *Labridae*: *Labrus bergylta* Ascanius; *Moronidae*: *Dicentrarchus labrax* L., *Morone saxatilis* Walbaum; *Myxinidae*: *Myxine glutinosa* L.; *Oxudercidae*: *Periophthalmodon schlosseri* Pallas; *Pleuronectidae*: *Platichthys flesus* L.; *Pomacentridae*: *Amphiprion clarkii* Bennet; *Salmonidae*: *Oncorhynchus mykiss* Walbaum, *Salvelinus alpinus* L., *Salvelinus fontinalis* Mitchill; *Sciaenidae*: *Argyrosomus regius* Asso, *Umbrina cirrosa* L.; *Scophthalmidae*: *Scophthalmus maximus* L., *Scophthalmus rhombus* L.; *Serranidae*: *Epinephelus marginatus* Lowe, *Epinephelus tauvina* Forsskål; *Soleidae*: *Solea senegalensis* Kaup, *Solea solea* L.; *Sparidae*: *Dentex dentex* L., *Pagellus bogaraveo* Brünnich, *Sparus aurata* L.

**Table 4:** List of antifungal studies using skin mucus from different fish species.

Fish Species (Family)	Extraction ^1^	Sensitive Fungi	Non-Sensitive Fungi	Antimicrobial Assay ^2^	Reference
*Anguilla anguilla* L. (Anguillidae)	C	*Aspergillus awamori*, *Colletotrichum falcatum*, *Fusarium oxysporum*		DD	[146]
*Catla catla* Hamilton (Cyprinidae)	PS	*Aspergillus flavus*, *Aspergillus niger*, *Candida albicans*, *Mucor globosus*, *Rhizopus arrhizus*		DD	[153]
	C	*A. awamori*, *C. falcatum*, *F. oxysporum*		DD	[146]
*Cirrhinus mrigala* Hamilton (Cyprinidae)	C	*A. awamori*, *C. falcatum*, *F. oxysporum*		DD	[146]
*Clarias batrachus* L. (Clariidae)	PS	*A. niger*, *Aspergillus nidulans*, *Fusarium moniliforme*, *C. albicans*, *Trichoderma koningi*		DD	[149]
*Ctenopharyngodon idella* Valenciennes (Cyprinidae)	PS	*A. flavus*, *C. albicans*, *M. globosus*, *R. arrhizus*	*A. niger*	DD	[153]
*Cyprinus carpio* L. (Cyprinidae)	PS, AA, DCM	*C. albicans*		BD	[140]
*Dasyatis pastinaca* L. (Dasyatidae)	C	*C. albicans*, *Candida glabrata*, *C. tropicalis*		BD	[158]
*Gadus morhua* L. (Gadidae)	ACN + 1% TFA	*C. albicans*		AWD, BD	[76]
	W, DCM	*C. albicans*, *Candida brusei*, *C. tropicalis*, *Issatchenkia orientalis*, *Saccharomyces cerevisiae*		AWD, BD	[138]
*Hypophthalmichthys molitrix* Valenciennes (Cyprinidae)	PS	*A. flavus*, *A. niger*, *M. globosus*	*C. albicans*, *R. arrhizus*	DD	[153]
*Labeo rohita* Hamilton (Cyprinidae)	NaOH, NaOH + TH		*C. albicans*	AWD	[166]
	PS	*A. flavus*, *A. niger*, *C. albicans*, *M. globosus*, *R. arrhizus*		DD	[153]
*Melanogrammus aeglefinus* L. (Gadidae)	AB, AA, ET, DCM	*C. albicans*		BD	[140]
*Monopterus albus* Zuiew (Synbranchidae)	C, W, PS	*C. albicans*, *Candida krusei*, *Cryptococcus neoformans*, *Fusarium* sp.		DD	[167]
*Morone saxatilis* Walbaum (Moronidae)	AB, AA, ET, DCM	*C. albicans*		BD	[140]
*Myxine glutinosa* L. (Myxinidae)	AB, AA, ET, DCM	*C. albicans*		BD	[140]
*Oncorhynchus mykiss* Walbaum (Salmonidae)	C		*C. albicans*, *Candida parapsilosis*	DD	[159]
*Periophthalmodon schlosseri* Pallas (Gobiidae)	PS, ET	*C. albicans*, *A. flavus*, *Mucor* sp., *Trichoderma longibriachtin*		DD, BD	[145]
*Salvelinus alpinus* L. (Salmonidae)	AB, AA, ET, DCM	*C. albicans*		BD	[140]
*Salvelinus fontinalis* Mitchill (Salmonidae)	AB, AA, ET, DCM	*C. albicans*		BD	[140]
*Scophtalamus rhombus* L. (Scophthalmidae)	W, ET, DCM	*C. tropicalis*, *S. cerevisiae*	*C. brusei*, *C. albicans*, *I. orientalis*	AWD, BD	[138]

^1^ C: crude; PS: physiological saline; AA: acetic acid; DCM: dichloromethane; ACN: acetonitrile; TFA: trifluoroacetic acid; W: water; AB: ammonium bicarbonate; ET: ethanol; TH: tris hydrochloride; ^2^ DD: disc diffusion; BD: broth dilution; AWD: agar-well diffusion.

## Data Availability

Not applicable.

## Supplementary Materials

The following supporting information can be downloaded at: https://www.mdpi.com/article/10.3390/md21060350/s1, Table S1: List of antibacterial studies using aqueous skin mucus extracts from different fish species; Table S2: List of antibacterial studies using organic skin mucus extracts from different fish species; Table S3: List of antibacterial studies using acidic skin mucus extracts from different fish species; Table S4: List of antibacterial studies using crude skin mucus from different fish species. References [126,127,128,129,130,131,132,133,134,135,136,137,138,139,140,141,142,143,144,145,146,147,148,149,150,151,152,153,154,155,156,157,158,159] are cited in the Supplementary Materials.
