# Supplementary material for: Fish Skin Mucus Extracts: An Underexplored Source of Antimicrobial Agents
Source: Mar Drugs. 2023 Jun 7;21(6):350. doi: 10.3390/md21060350 (PMC10303038; doi:10.3390/md21060350)
Supplement: Supplementary file 1 [file marinedrugs-21-00350-s001.zip › marinedrugs-2421954-supplementary.pdf]

marine drugs

Review

Fish Skin Mucus Extracts: An Underexplored Source of Antimicrobial Agents

Rocío Díaz-Puertas [1], Mikolaj Adamek [2], Ricardo Mallavia [1] and Alberto Falco [1,*]

[1] Institute of Research, Development and Innovation in Healthcare Biotechnology in Elche (IDiBE), Miguel Hernández University, 03202 Elche, Spain; r.diaz@umh.es (R.D.-P.); r.mallavia@umh.es (R.M.)
[2] Fish Disease Research Unit, Institute for Parasitology, University of Veterinary Medicine, 30559 Hannover, Germany; mikolaj.adamek@tiho-hannover.de
* Correspondence: alber.falco@umh.es

Supplementary Materials

Summary:

Table S1: List of antibacterial studies using aqueous skin mucus extracts from different fish species.

Table S2: List of antibacterial studies using organic skin mucus extracts from different fish species.

Table S3: List of antibacterial studies using acidic skin mucus extracts from different fish species.

Table S4: List of antibacterial studies using crude skin mucus from different fish species.

Table S1. List of antibacterial studies using aqueous skin mucus extracts from different fish species.

Fish species	Extraction [1]	Bacteria [2]		Antimicrobial assay [3]	Ref.
		Sensitive	Resistant		
Amphiprion clarkii	W	G-: *Aeromonas hydrophila, Pseudomonas fluorescens, Vibrio alginolyticus, Vibrio harveyi, Vibrio parahaemolyticus*	G+: *Micrococcus lysodeikticus, Staphylococcus aureus*	BD	[1]
Anabas testudineus	AB		G+: *Bacillus subtilis, S. aureus* G-: *A. hydrophila, Escherichia coli, Pseudomonas aeruginosa, Salmonella spp., Salmonella choleraesuis, Serratia marcescens*	AWD	[2]
Anguilla anguilla	PS	G+: *S. aureus,* G-: *V. alginolyticus, Vibrio fluvialis, V. parahaemolyticus*	G+: *Enterococcus faecium, Staphylococcus epidermidis* G-: *A. hydrophila, E. coli, Klebsiella pneumoniae, Photobacterium damselae* subsp. *piscicida, P. aeruginosa, Salmonella typhi, Vibrio anguillarum*	DD	[3]
Arius maculatus	PS	G-: *E. coli, P. aeruginosa, Salmonella spp., Shigella spp., Vibrio cholerae*		DD	[4]
Barbonymus schwanenfeldii	W		G+: *Bacillus cereus, S. aureus* G-: *Shigella boydii, E. coli*	DD	[5]
Catla catla	PS	G-: *A. hydrophila, Aeromonas sobria, P. fluorescens, V. anguillarum.*		DD	[6]
	PS	G-: *E. coli, K. pneumoniae, P. aeruginosa, S. typhi, V. cholerae*		DD	[7]
Channa gachua	PS	G-: *A. hydrophila, E. coli, P. aeruginosa, V. anguillarum, Vibrio fischeri*		DD	[8]
Channa marulius	PS	G-: *A. hydrophila, E. coli, P. aeruginosa, V. anguillarum, V. fischeri*		DD	[8]
Channa micropeltes	PS	G-: *A. hydrophila, E. coli, P. aeruginosa, V. anguillarum, V. fischeri*		DD	[8]
Channa punctatus	PS	G+: *Lactobacillus bulgaricus, S. aureus* G-: *E. coli, Klebsiella oxytoca, K. pneumoniae, P. aeruginosa,*		DD	[9]

		Proteus mirabilis, S. paratyphi, S. typhi, V. cholerae			
	PS	G-: *A. hydrophila, E. coli, P. aeruginosa, V. anguillarum, V. fischeri*		DD	[8]
	W	G+: *S. aureus, Micrococcus luteus*	G -: *E. coli, P. aeruginosa, S. typhi*	DD	[10]
Channa striatus	W	G-: *A. hydrophila*	G+: *B. subtilis* G-: *K. pneumoniae, P. aeruginosa, Proteus vulgaris, Salmonella enteritidis*	DD, BD	[11]
	W	G+: *L. bulgaricus, S. aureus* G-: *E. coli, K. oxytoca, P. aeruginosa, K. pneumoniae, S. paratyphi, S. typhi, P. mirabilis, V. cholerae*		AWD	[12]
	PS	G+: *B. subtilis, M. luteus, S. aureus, Steptococcus pyogenes* G-: *E. coli, P. vulgaris, P. aeruginosa, Salmonella typhimurium, V. cholera, Mycobacterium smegmatis*		AWD	[13]
	PS	G-: *A. hydrophila, E. coli, P. aeruginosa, V. anguillarum, V. fischeri*		DD	[8]
Cirrhinus mrigala	PS	G+: *L. bulgaricus S. aureus* G-: *E. coli, K. oxytoca, K. pneumoniae, P. aeruginosa, P. mirabilis, S. paratyphi, S. typhi, V. cholerae*		DD	[9]
	W	G-: *P. aeruginosa, S. paratyphi, S. typhi, V. cholerae*	G+: *S. aureus,*	DD	[14]
Clarias batrachus	AB	G+: *S. aureus* G-: *A. hydrophila, E. coli, K. pneumonia, P. aeruginosa, P. vulgaris*	G+: *Bacillus coagulans*	DD	[15]
	PS	G-: *A. hydrophila, E. coli, P. aeruginosa, V. anguillarum, V. fischeri*		DD	[16]
Ctenopharyngodon idella	PS	G+: *B. cereus, S. aureus, S. epi-dermidis* G-: *A. hydrophila, E. coli, K. pneumoniae, P. aeruginosa*		AWD	[17]

	PS	G-: *A. hydrophila, A. sobria, P. fluorescens, V. anguillarum.*		DD	[6]
	PS	G-: *E. coli, K. pneumoniae, P. aeruginosa, S. typhi, V. cholerae*		DD	[7]
Cyprinus carpio	AB		G+: *S. epidermis*<br>G-: *Aeromonas salmonicida, E. coli, Listonella anguillarum, P. aeruginosa, Salmonella enterica, Yersinia ruckeri*	BD	[18]
	PS	G+: *S. epidermis*<br>G-: *A. salmonicida, E. coli, L. anguillarum, P. aeruginosa, S. enterica, Y. ruckeri*		AWD	[17]
Dentex dentex	TBS	G+: *B. subtilis*<br>G-: *E. coli, P. damselae, Shewanella putrefaciens, V. harveyi, V. angillarum*		OD	[19]
Dicentrarchus labrax	PS	G+: *S. aureus*	G-: *P. damselae* subsp. *piscicida, Tenacibaculum maritimum, V. anguillarum, V. damsela*	DD	[20]
	PS	G+: *S. aureus*<br>G-: *V. alginolyticus, V. fluvialis, V. parahaemolyticus*	G+: *E. faecium, S. epidermidis*<br>G-: *A. hydrophila, E. coli, K. pneumoniae, P. damselae* subsp. *piscicida, P. aeruginosa, S. typhi, V. anguillarum,*	DD	[3]
	TBS	G+: *B. subtilis*<br>G-: *E. coli, P. damselae, S. putrefaciens, V. harveyi, V. angillarum*		OD	[19]
Epinephelus marginatus	TBS	G+: *B. subtilis*<br>G-: *E. coli, P. damselae, S. putrefaciens, V. harveyi, V. angillarum*		OD	[19]
Epinephelus tauvina	AB		G-: *A. hydrophila, E. coli, S. typhi, K. pneumonia, P. mirabilis, P. fluorescens, V. alginolyticus, V. harveyi, V. parahemolyticus*	AWD	[21]
Heteropneustes fossilis	PS	G+: *B. subtilis, M. luteus, S. aureus, S. pyogenes*<br>G-: *E. coli, P. vulgaris, P. aeruginosa, S. typhimurium, V. cholera, M. smegmatis*		AWD	[13]
Hypophthalmichthys nobilis	PS	G+: *B. cereus, S. aureus, S. epidermidis*		AWD	[17]

		G-: *A. hydrophila, E. coli, K. pneumoniae, P. aeruginosa*			
	PS	G-: *A. hydrophila, A. sobria, P. fluorescens, V. anguillarum.*		DD	[6]
	PS	G-: *E. coli, K. pneumoniae, P. aeruginosa, S. typhi, V. cholerae*		DD	[7]
	PS	G+: *B. cereus, S. aureus, S. epidermidis*<br>G-: *A. hydrophilla, E. coli, K. pneumonia, P. aeruginosa,*		AWD, APD	[22]
Labeo rohita	PS	G-: *A. hydrophila, A. sobria, P. fluorescens, V. anguillarum.*		DD	[6]
	PS	G-: *E. coli, K. pneumoniae, P. aeruginosa, S. typhi, V. cholerae*		DD	[7]
Labrus bergylta	W		G+: *B. cereus, B. subtilis, Bacillus megaterium, S. aureus, Streptococcus sp.*<br>G-: *E. coli, K. pneumoniae, P. vulgaris, P. aeruginosa, S. marcescens*	AWD, BD	[23]
	W		G+: *B. subtilis, Lactobacillus plantarum*<br>G-: *A. sobria, Citrobacter sp., Edwardsiella tarda, E. coli, Enterobacter sp., L. anguillarum, Shewanella baltica, Y. ruckeri*	DD	[24]
Melanogrammus aeglefinus	AB		G+: *S. epidermis*<br>G-: *A. salmonicida, E. coli, L. anguillarum, P. aeruginosa, S. enterica, Y. ruckeri*	BD	[18]
Morone saxatilis	AB		G+: *S. epidermis*<br>G-: *A. salmonicida, E. coli, L. anguillarum, P. aeruginosa, S. enterica, Y. ruckeri*	BD	[18]
Myxine glutinosa	AB		G-: *S. enterica*	BD	[18]
Oreochromis niloticus	W		G-: *V. harveyi, V. parahaemolyticus*	BD, OD	[25]
Oreochromis mossambicus	AB	G+: *S. aureus*<br>G-: *A. hydrophila, E. coli, K. pneumonia, P. aeruginosa, P. vulgaris*	G+: *B. coagulans*	DD	[15]
Pagellus bogaraveo	PS	G-: *V. parahaemolyticus*	G+: *E. faecium, S. aureus, S. epidermidis*<br>G-: *A. hydrophila, E. coli, K. pneumoniae, P. damselae* subsp.	DD	[3]

			iscicida, P. aeruginosa, S. typhi, V. alginolyticus, V. anguillarum, V. fluvialis		
Periophthalmodon schlosseri	PS	G+: *Bacillus anthracis, S. aureus* G-: *E. coli, P. mirabilis, P. aeruginosa, S. typhi, V. cholerae, K. pneumoniae*		DD, BD	[26]
Platichthys flesus	W	G+: *B. cereus, B. subtilis, B. megaterium, S. aureus, Streptococcus sp.* G-: *E. coli, K. pneumoniae, P. vulgaris, P. aeruginosa, S. marcescens*		AWD, BD	[23]
Pollachius virens	W	G+: *B. cereus, B. subtilis, B. megaterium, S. aureus, Streptococcus sp.* G-: *E. coli, K. pneumoniae, P. vulgaris, P. aeruginosa, S. marcescens*		AWD, BD	[23]
Rita rita	W	G+: *S. aureus, M. luteus* G -: *S. typhi*	G -: *E. coli, P. aeruginosa*	DD	[10]
Salvelinus alpinus	AB		G+: *S. epidermis* G-: *A. salmonicida, E. coli, L. anguillarum, P. aeruginosa, S. enterica, Y. ruckeri*	BD	[18]
Salvelinus fontinalis	AB		G+: *S. epidermis* G-: *A. salmonicida, E. coli, L. anguillarum, P. aeruginosa, S. enterica, Y. ruckeri*	BD	[18]
Scaphtalamus rhombus	W	G+: *B. cereus, B. subtilis, B. megaterium, S. aureus, Streptococcus sp.* G-: *E. coli, K. pneumoniae, P. vulgaris, P. aeruginosa, S. marcescens*		AWD, BD	[23]
Scophthalamus maximus	PS	G+: *S. aureus* G-: *P. damselae* subsp. *piscicida, V. anguillarum*	G-: *T. maritimum, V. damsela*	DD	[20]
Solea senegalensis	TBS	G+: *B. subtilis* G-: *E. coli, P. damselae subsp. piscicida, S. putrefaciens, V. anguillarum, V. harveyi*		OD	[27]

Solea solea	W	G+: *B. cereus, B. subtilis, B. megaterium, S. aureus, Streptococcus sp.*<br>G-: *E. coli, K. pneumoniae, P. vulgaris, P. aeruginosa, S. marcescens*		AWD, BD	[23]
Sparus aurata	PS	G+: *S. aureus*	G-: *P. damselae* subsp. *piscicida, T. maritimum, V. anguillarum, V. damsela*	DD	[20]
	TBS	G+: *B. subtilis*<br>G-: *E. coli, P. damselae, S. putrefaciens, V. harveyi, V. angillarum*		OD	[19]
Umbrina cirrosa	TBS	G+: *B. subtilis*<br>G-: *E. coli, P. damselae, S. putrefaciens, V. harveyi, V. angillarum*		OD	[19]

[1] AB: ammonium bicarbonate; PS: physiological saline; W: water; TBS: tris buffered saline

[2] G+: gram-positive; G-: gram-negative

[3] APD: agar plate dilution; AWD: agar well diffusion; BD: broth dilution; DD: disc diffusion; OD: optical density

Table S2. List of antibacterial studies using organic skin mucus extracts from different fish species.

Fish species	Extraction [1]	Bacteria [2]		Antimicrobial assay [3]	Ref.
		Sensitive	Resistant		
Barbonymus Schwanenfeldii	ET, DCM	G+: *Bacillus cereus, Staphylococcus aureus* G-: *Shigella boydii, Escherichia coli*		DD	[5]
Cyprinus carpio	DCM	G-: *Salmonella enterica*		BD	[18]
Epinephelus tauvina	ET		G-: *Aeromonas hydrophila, E. coli, Salmonella typhi, Klebsiella pneumonia, Proteus mirabilis, Pseudomonas fluorescens, Vibrio alginolyticus, Vibrio harveyi, Vibrio parahemolyticus*	AWD	[21]
Gadus morhua	ACN + 1% TFA	G+: *Bacillus megaterium* G-: *E. coli*		AWD, BD	[28]
Labrus bergylta	DCM	G-: *E. coli, K. pneumoniae, Proteus vulgaris, Pseudomonas aeruginosa, Serratia marcescens*	G+: *B. cereus, Bacillus subtilis, B. megaterium, S. aureus, Streptococcus sp.*	AWD, BD	[23]
	DCM		G+: *B. subtilis, Lactobacillus plantarum* G-: *Aeromonas sobria, Citrobacter sp., Edwardsiella tarda, E. coli, Enterobacter sp., Listonella anguillarum, Shewanella baltica, Yersinia ruckeri*	DD	[24]
Melanogrammus aeglefinus	DCM		G-: *S. enterica*	BD	[18]
Morone saxatilis	DCM	G-: *S. enterica*		BD	[18]
Myxine glutinosa	DCM		G-: *S. enterica*	BD	[18]
Oreochromis niloticus	DCM	G-: *V. harveyi*	G-: *V. parahaemolyticus*	BD, OD	[25]
	ET	G-: *V. harveyi*		DD, BD	[29]
Periophthalmodon schlosseri	ET	G+: *Bacillus anthracis, S. aureus* G-: *E. coli, P. mirabilis, P. aeruginosa, S. typhi, Vibrio cholerae, K. pneumoniae*		DD, BD	[26]
Platichthys flesus	DCM	G+: *B. cereus, B. subtilis, B. megaterium, S. aureus, Streptococcus sp.* G-: *E. coli, K. pneumoniae, P. vulgaris,*		AWD, BD	[23]

		P. aeruginosa, S. marcescens			
Pollachius virens	DCM	G+: *B. cereus, B. subtilis, B. megaterium, S. aureus, Streptococcus sp.* G-: *E. coli, K. pneumoniae, P. vulgaris, P. aeruginosa, S. marcescens*		AWD, BD	[23]
Salvelinus alpinus	DCM		G-: *S. enterica*	BD	[18]
Salvelinus fontinalis	DCM	G-: *S. enterica*		BD	[18]
Scaphtalamus rhombus	DCM	G+: *B. cereus, B. subtilis, B. megaterium, S. aureus, Streptococcus sp.* G-: *E. coli, K. pneumoniae, P. vulgaris, P. aeruginosa, S. marcescens*		AWD, BD	[23]
Solea solea	DCM	G+: *B. cereus, B. subtilis, B. megaterium, S. aureus, Streptococcus sp.* G-: *E. coli, K. pneumoniae, P. vulgaris, P. aeruginosa, S. marcescens*		AWD, BD	[23]

[1] ACN: acetonitrile; DCM: dichloromethane; ET: ethanol; TFA: trifluoroacetic acid.

[2] G+: gram-positive; G-: gram-negative

[3] AWD: agar well diffusion; BD: broth dilution; DD: disc diffusion; OD: optical density

Table S3. List of antibacterial studies using acidic skin mucus extracts from different fish species.

Fish species	Extraction [1]	Bacteria [2]		Antimicrobial assay [3]	Ref.
		Sensitive	Resistant		
Anabas testudineus	AA	G+: *Bacillus subtilis, Staphylococcus aureus* G-: *Aeromonas hydrophila, Escherichia coli, Pseudomonas aeruginosa, Salmonella spp., Salmonella choleraesuis, Serratia marcescens*		AWD	[2]
Channa punctatus	AA, TFA	G+: *S. aureus, Micrococcus luteus*	G -: *E. coli, P. aeruginosa, Salmonella typhi*	DD	[10]
Channa striatus	AA	G+: *B. subtilis* G-: *A. hydrophila, Klebsiella pneumoniae, P. aeruginosa, Proteus vulgaris, Salmonella enteritidis*		DD, BD	[11]
	AA	G+: *Enterococcus faecalis, M. luteus, S. aureus,* G-: *A. hydrophila, E. coli, K. pneumoniae, P. aeruginosa*		AWD	[30]
Cirrhinus mrigala	AA	G+: *S. aureus,* G-: *P. aeruginosa, S. paratyphi, S. typhi, V. cholerae*		DD	[14]
	TFA	G-: *S. paratyphi, V. cholerae*	G+: *S. aureus,* G-: *P. aeruginosa, S. typhi*	DD	[14]
Clarias batrachus	AA	G+: *E. faecalis, M. luteus, S. aureus* G-: *A. hydrophila, E. coli, K. pneumoniae, P. aeruginosa*		AWD	[30]
Cyprinus carpio	AA		G+: *Staphylococcus epidermis* G-: *Aeromonas salmonicida, E. coli, Listonella anguillarum, P. aeruginosa, Salmonella enterica, Yersinia ruckeri*	BD	[18]
Epinephelus tauvina	AA	G-: *A. hydrophila, E. coli, S. typhi, K. pneumonia, Proteus mirabilis, Pseudomonas fluorescens, Vibrio alginolyticus, Vibrio harveyi, Vibrio parahemolyticus*		AWD	[21]
Melanogrammus aeglefinus	AA	G+: *S. epidermis* G-: *A. salmonicida, E. coli, L. anguillarum, P. aeruginosa, S. enterica, Y. ruckeri*		BD	[18]
Morone saxatilis	AA		G-: *S. enterica*	BD	[18]

Myxine glutinosa	AA	G+: *S. epidermis*<br>G-: *A. salmonicida, E. coli, L. anguillarum, P. aeruginosa, S. enterica, Y. ruckeri*		BD	[18]
Oreochromis niloticus	AA	G-: *V. harveyi*	G-: *V. parahaemolyticus*	BD, OD	[25]
	AA	G+: *E. faecalis, M. luteus, S. aureus,*<br>G-: *A. hydrophila, E. coli, K. pneumoniae, P. aeruginosa*		AWD	[30]
Puntius sophore	AA	G+: *B. subtilis, S. aureus*<br>G-: *E. coli, P. aeruginosa*		AWD	[31]
Rita rita	AA, TFA	G+: *S. aureus and M. luteus*<br>G -: *S. typhi*	G -: *E. coli, P. aeruginosa*	DD	[10]
Salvelinus alpinus	AA		G-: *S. enterica*	BD	[18]
Salvelinus fontinalis	AA	G+: *S. epidermis*<br>G-: *A. salmonicida, E. coli, L. anguillarum, P. aeruginosa, Salmonella enterica, Y. ruckeri*		BD	[18]

[1] AA: acetic acid TFA: trifluoroacetic acid.

[2] G+: gram-positive; G-: gram-negative

[3] AWD: agar well diffusion; BD: broth dilution; DD: disc diffusion; OD: optical density

Table S4. List of antibacterial studies using crude skin mucus from different fish species.

Fish species	Bacteria [1]		Antimicrobial assay [2]	Ref.
	Sensitive	Resistant		
Argyrosomus regius	G-: *Escherichia coli, Pseudomonas anguilliseptica, Vibrio anguillarum*		OD	[32]
Channa argus	G-: *E. coli*		OD	
Channa striatus	G-: *A. hydrophila*	G+: *Bacillus subtilis*<br>G-: *Klebsiella pneumoniae, Pseudomonas aeruginosa, Proteus vulgaris, Salmonella enteritidis*	DD, BD	[11]
Clarias batrachus	G+: *B. subtilis, Staphylococcus aureus*<br>G-: *K. pneumoniae, P. vulgaris, P. aeruginosa, S. paratyphi*		DD	[33]
Ctenopharyngodon idella	G+: *Bacillus cereus, S. aureus, Staphylococcus epidermidis*<br>G-: *A. hydrophila, E. coli, K. pneumoniae, P. aeruginosa*		AWD	[17]
Cyprinus carpio	G+: *B. cereus, S. aureus, S. epidermidis*<br>G-: *A. hydrophila, E. coli, K. pneumoniae, P. aeruginosa*		AWD	[17]
Dasyatis pastinaca	G-: *E. coli, K. pneumoniae, P. aeruginosa*	G+: *E. faecalis, S. aureus, Streptococcus agalactiae*	CC	[34]
Dicentrarchus labrax	G-: *E. coli, V. anguillarum*	G-: *Pseudomonas anguilliseptica*	OD	[32]
Oncorhynchus mykiss		G+: *B. cereus, S. aureus, S. pneumoniae*<br>G-: *Citrobacter freundii, Enterobacter aerogenes, E. coli, Klebsiella oxyttoca, K. pneumoniae, Neisseria lactamica, Proteus mirabilis, Pseudomonas fluorescens, P. vulgaris, P. aeruginosa*	DD	[35]
Sparus aurata	G-: *E. coli, Pseudomonas anguilliseptica, V. anguillarum,*		OD	[32]

[1] G+: Gram-positive; G-: Gram-negative

[2] AWD: agar well diffusion; BD: broth dilution; CC: cell counting; DD: disc diffusion; OD: optical density

References

1. Wang, H.; Tang, W.; Zhang, R.; Ding, S., Analysis of enzyme activity, antibacterial activity, antiparasitic activity and physico-chemical stability of skin mucus derived from Amphiprion clarkii. *Fish & Shellfish Immunology* **2019,** 86, 653-661.
2. Al-Rasheed, A.; Handool, K. O.; Garba, B.; Noordin, M. M.; Bejo, S. K.; Kamal, F. M.; Daud, H. H. M., Crude extracts of epidermal mucus and epidermis of climbing perch A*nabas testudineus* and its antibacterial and hemolytic activities. *The Egyptian Journal of Aquatic Research* **2018,** 44, (2), 125-129.
3. Caruso, G.; Maricchiolo, G.; Genovese, L.; Pasquale, F. D.; Rosalba; Caruso; Denaro, M. G.; Delia, S.; Laganà, P., Comparative Study of Antibacterial and Haemolytic Activities in Sea Bass, European Eel and Blackspot Seabream. *The Open Marine Biology Journal* **2014,** 8, 10-16.
4. Manivasagan, P.; Annamalai, N.; Ashokkumar, S.; Sampathkumar, P., Studies on the proteinaceous gel secretion from the skin of the catfish, *Arius maculatus* (Thunberg, 1792). *African Journal of Biotechnology* **2009,** 8, (24), 7125-7129.
5. Subhashini, S.; Lavanya, J.; Jain, S.; Agihotri, T., Screening of Antibacterial and Cytotoxic Activity of Extracts From Epidermis and Epidermal Mucus of B*arbonymus Schwanenfeldii* (Tinfoil Barb Fish). *International Journal of Research in Engineering and Technology* **2013,** 2, (4), 492-497.
6. Islam, M. M.; Hossain, M. M. M.; Islam, M. S.; Shoumo; Khondoker; Khatun, M. A., Competitive antibacterial activity of two Indian majorcarps and two Chinese carps fish mucus against common pathogenic bacteria at aquaculture pond. *International Journal of Fisheries and Aquatic Studies* **2014,** 2, (2), 158-162.
7. Balasubramanian, S.; Rani, P. B.; Prakash, A. A.; Prakash, M.; Senthilraja, P.; Gunasekaran, G., Antimicrobial properties of skin mucus from four freshwater cultivable Fishes (*Catla catla*, *Hypophthalmichthys molitrix*, *Labeo rohita* and *Ctenopharyngodon idella*). *African Journal of Microbiology Research* **2011,** 6, (24), 5110-5120.
8. Dhanaraj, M.; Haniffa, M. A.; Singh, S. V. A.; Ramakrishnan, C. M.; Manikandaraja, D.; Milton, M. J., Antibacterial Activity of Skin and Intestinal Mucus of Five Different Freshwater Fish Species Viz., *Channa striatus*, *C. micropeltes*, *C. marulius*, *C. punctatus* and *C. gachua*. *Malaysian Journal of Science* **2009,** 28, (3), 257-262.
9. Kuppulakshmi, C.; Prakash, M.; Gunasekaran, G.; Manimegalai, G.; Sarojini, S., Antibacterial properties of fish mucus from Channa punctatus and Cirrhinus mrigala. *European Review of Medical Pharmacological Science* **2008,** 12, (3), 149-53.
10. Kumari, U.; Nigam, A. K.; Mittal, S.; Mittal, A. K., Antibacterial properties of the skin mucus of the freshwater fishes, *Rita rita* and *Channa punctatus*. *European Review for Medical and Pharmacological Sciences* **2011,** 15, 781-786.
11. Wei, O. Y.; Xavier, R.; Marimuthu, K., Screening of antibacterial activity of mucus extract of snakehead fish, *Channa striatus*. *European Review for Medical and Pharmacological Sciences* **2010,** 14, 675-681.
12. Ramesh, B., Assessment of Antimicrobial peptides from mucus of fish. *International Journal of Current Biotechnology* **2013,** 1, (1), 5-8.
13. Haniffa, M. A.; Viswanathan, S.; Jancy, D.; Poomari, K.; Manikandan, S., Antibacterial studies of fish mucus from two marketed air-breathing fishes – *Channa striatus* and *Heteropneustes fossilis*. *International Research Journal of Microbiology* **2014,** 5, (2), 22-27.
14. Nigam, A. K.; Kumari, U.; Mittal, S.; Mittal, A. K., Evaluation of antibacterial activity and innate immune components in skin mucus of Indian major carp, *Cirrhinus mrigala*. *Aquaculture Research* **2015,** 48, (2), 407-418.
15. Elavarasi, K.; Ranjini, S.; Rajagopal, T.; Rameshkumar, G.; Ponmanickam, P., Bactericidal proteins of skin mucus and skin extracts from fresh water fishes, C*larias batrachus a*nd T*ilapia mossambicus Thai Journal of Pharmaceutical Sciences* **2013,** 37, (1), 194-200.
16. Loganathan, K.; Muniyan, M.; Prakash, A. A.; Raja, P. S.; Prakash, M., Studies on the Role of Mucus From C*larias batrachus* (Linn) Against Selected Microbes. *International Journal of Pharmaceutical Applications* **2011,** 2, (3), 202-206.
17. Kumari, S.; Tyor, A. K.; Bhatnagar, A., Evaluation of the antibacterial activity of skin mucus of three carp species. *International Aquatic Research* **2019,** 11, 225-239.

18. Subramanian, S.; Ross, N. W.; MacKinnon, S. L., Comparison of antimicrobial activity in the epidermal mucus extracts of fish. *Comparative Biochemistry and Physiology Part B: Biochemistry and Molecular Biology* **2008,** 150, (1), 85-92.
19. Guardiola, F. A.; Cuesta, A.; Abellán, E.; Meseguer, J.; Esteban, M. A., Comparative analysis of the humoral immunity of skin mucus from several marine teleost fish. *Fish & Shellfish Immunology* **2014,** 40, (1), 24-31.
20. Magarinos, B.; Pazos, F.; Santos, Y.; Romalde, J. L.; Toranzo, A. E., Response of *Pasteurella piscicida* and *Flexibacter maritimus* to skin mucus of marine fish. *Diseases of Aquatic Organisms* **1995,** 21, 103-108.
21. Manikantan, G.; Lyla, S.; Khan, S. A.; Vijayanand, P.; Jothi, G. E. G., Bioactive potency of epidermal mucus extracts from greasy grouper, Epinephelus tauvina (Forsskal, 1775). *Journal of Coastal Life Medicine* **2016,** 4, (7), 510-520.
22. Tyor, A. K.; Kumari, S., Biochemical characterization and antibacterial properties of fish skin mucus of fresh water fish, *Hypophthalmichthys nobilis*. *International Journal of Pharmacy and Pharmaceutical Sciences* **2016,** 8, (6), 132-136.
23. Hellio, C.; Pons, A. M.; Beaupoil, C.; Bourgougnon, N.; Gal, d. s. Y. L., Antibacterial, antifungal and cytotoxic activities of extracts from fish epidermis and epidermal mucus. *International Journal of Antimicrobial Agents* **2002,** 20, 214-219.
24. Katra, N.; Hisar, O.; Yilmaz, S.; Turgay, E.; Sarvan, C.; Suheyla; Karatas, *In vitro* antimicrobial activities of extracts from ballan wrasse (*Labrus bergylta*) skin mucus. *Marine Science and Technology Bulletin* **2016,** 5, (1), 13-15.
25. García-Marciano, M.; Apún-Molina, J. P.; Sainz-Hernández, J. C.; Santamaría-Miranda, A.; Medina-Godoy, S.; Aguiñaga-Cruz, J. A., Antibacterial activity evaluation of the Nile tilapia O*reochromis niloticus* (Linnaeus, 1758) skin mucus, against V*ibrio* bacteria affecting the white shrimp P*enaeus vannamei*. *Latin American Journal of Aquatic Research* **2019,** 47, (3), 580-585.
26. Mahadevan, G.; Mohan, K.; Vinoth, J.; Ravi, V., Biotic potential of mucus extracts of giant mudskipper Periophthalmodon schlosseri (Pallas, 1770) from Pichavaram, southeast coast of India. *The Journal of Basic and Applied Zoology* **2019,** 80, (1), 13.
27. Guardiola, F. A.; Cuartero, M.; Collado-González, M. d. M.; Díaz-Baños, F. G.; Cuesta, A.; Moriñigo, M. Á.; Esteban, M. Á., Terminal carbohydrates abundance, immune related enzymes, bactericidal activity and physico-chemical parameters of the Senegalese sole (S*olea senegalensis,* Kaup) skin mucus. *Fish & Shellfish Immunology* **2017,** 60, 483-491.
28. Bergsson, G.; Agerberth, B.; Jörnvall, H.; Gudmundsson, G. H., Isolation and identification of antimicrobial components from the epidermal mucus of Atlantic cod (*Gadus morhua*). *The FEBS Journal* **2005,** 272, 4960–4969.
29. Wibowo, A.; Fadjar, M.; Maftuch, Utilization of Tilapia Mucus to Inhibit *Vibrio harveyi* on Vannamei (*Litopenaeus vannamei*). *Journal of Life Science and Biomedicine* **2015,** 5, (5), 141-148.
30. Lirio, G. A. C.; Leon, J. A. A. D.; Villafuerte, A. G., Antimicrobial Activity of Epidermal Mucus from Top Aquaculture Fish Species against Medically-Important Pathogens. *Walailak Journal* **2018,** 16, (5), 329-340.
31. Patel, M.; Ashraf, M. S.; Siddiqui, A. J.; Ashraf, S. A.; Sachidanandan, M.; Snoussi, M.; Adnan, M.; Hadi, S., Profiling and Role of Bioactive Molecules from P*untius sophore (*Freshwater/Brackish Fish) Skin Mucus with Its Potent Antibacterial, Antiadhesion, and Antibiofilm Activities. *Biomolecules* **2020,** 10, (6).
32. Sanahuja, I.; Fernández-Alacid, L.; Ordóñez-Grande, B.; Sánchez-Nuño, S.; Ramos, A.; Araujo, R. M.; Ibarz, A., Comparison of several non-specific skin mucus immune defences in three piscine species of aquaculture interest. *Fish & Shellfish Immunology* **2019,** 89, 428-436.
33. Patil, R. N.; Kadam, J. S.; Ingole, J. R.; Sathe, T. V.; Jadhav, A. D., Antibacterial activity of fish mucus from *Clarias batrachus* (Linn.) against selected microbes. *Biolife* **2015,** 3, (4), 788-791.
34. Fuochi, V.; Volti, G. L.; Camiolo, G.; Tiralongo, F.; Giallongo, C.; Distefano, A.; Petronio, G. P.; Barbagallo, I.; Viola, M.; Furneri, P. M.; Rosa, M. D.; Avola, R.; Tibullo, D., Antimicrobial and Anti-Proliferative Effects of Skin Mucus Derived from *Dasyatis pastinaca*. *Marine drugs* **2017,** 15, (11).
35. Hisar, O.; Hisar, S. A.; Uyanik, M. H.; Sahin, T.; Cakir, F.; Yilmaz, S., *In vitro* antimicrobial and antifungal activities of aqueous skin mucus from rainbow trout (*Oncorhynchus mykiss*) on human pathogens. *Marine Science and Technology Bulletin* **2014,** 3, (1), 19-22.
